# Acute Effects of Whole-Body Vibration on Resting Metabolic Rate and Substrate Utilisation in Healthy Women

**DOI:** 10.3390/biology11050655

**Published:** 2022-04-24

**Authors:** Marcin Maciejczyk, Marek Bawelski, Magdalena Więcek, Zbigniew Szygula, Michail Lubomirov Michailov, Bibiana Vadašová, Peter Kačúr, Tomasz Pałka

**Affiliations:** 1 Department of Physiology and Biochemistry, Faculty of Physical Education and Sport, University of Physical Education, 31-571 Kraków, Poland; marek.bawelski@awf.krakow.pl (M.B.); magdalena.wiecek@awf.krakow.pl (M.W.); tomasz.palka@awf.krakow.pl (T.P.); 2 Department of Sports Medicine and Human Nutrition, Faculty of Physical Education and Sport, University of Physical Education, 31-571 Kraków, Poland; wfszygul@cyf-kr.edu.pl; 3 Department Theory and Methodology of Sports Training, National Sports Academy, 1700 Sofia, Bulgaria; michailovi@hotmail.com; 4 Department of Sports Kinanthropology, Faculty of Sports, University of Presov, 080 01 Presov, Slovakia; bibiana.vadasova@unipo.sk; 5 Department of Sports Educology and Humanistics, Faculty of Sports, University of Presov, 080 01 Presov, Slovakia; peter.kacur@unipo.sk

**Keywords:** metabolism, women, vibration, substrate utilization, oxygen uptake, energy, calorimetry

## Abstract

**Simple Summary:**

Whole-body vibration induces a number of physiological responses and is used both in physical training, and in the treatment of patients with various diseases. Whole-body vibration increases blood flow, skin temperature, bone density, muscle strength and affects the functions of the nervous system. The effect of whole-body vibration on metabolism is interesting. It has been suggested that whole-body vibration shifts energy metabolism towards carbohydrate utilisation and that this may increase post-exercise oxygen uptake. Increased resting metabolism (increased energy expenditure) during whole-body vibration may indicate that whole-body vibration can be used as an adjunct to treatment programs for overweight or metabolic disorders.

**Abstract:**

The aim of the study was to determine the acute effects of single-whole-body vibration (WBV) on resting metabolic rate (RMR) and carbohydrate–lipid profile of blood in young, healthy women. The participants, in a randomised controlled crossover study, participated in two trials: WBV and a vibration simulation (placebo). The WBV was performed in the prone position and cycloidal-oscillatory vibration was used. The RMR measurement (calorimetry) was performed: before the WBV, during WBV, immediately after the completion of WBV, and 1 h after the completion of WBV. For biochemical analyses, venous blood was collected. During WBV, there was a significant increase in RMR compared to baseline. Immediately after and 1 h following the end of the WBV, RMR was close to baseline levels (*p* > 0.05). The increased energy expenditure resulted from the increased utilisation of carbohydrates and proteins during the vibration. In the placebo condition, there were no significant changes over time in the level of the studied indices during calorimetry. The WBV had no significant effects on the level of glucose in the blood. The applied vibration did not significantly affect the concentration of the analysed lipid indices, which were within the physiological norms for all measurements. Results indicate the need for further research to establish the physiological mechanisms underlying the observed effects of WBV on resting metabolic rate.

## 1. Introduction

Whole-body vibration (WBV) induces a number of physiological responses and is used both in physical training [1], e.g., in reducing muscle damage [2], and in the treatment of patients with various diseases, e.g., osteoporosis [3], sarcopenia [4], or metabolic syndrome [5]. In previous studies, it has been shown that the use of vibration causes, among others, such physiological effects as: pain decrease [6], muscle activation [7,8], changes in blood flow [9]—in particular an increase in cutaneous blood flow [10]—changes in skin temperature [11], or effects on the functions of the nervous system [11]. Vibration appears to elicit an alteration in neuromuscular recruitment patterns, which apparently enhance neuromuscular excitability [12]. Bone mass density and isometric leg strength improvements were also reported [13]. Due to the growing interest in this type of exercise/therapy, there is a need for further studies [14,15], that would take into account various types of vibration, body position, or assessment of the effects of WBV in various populations (age, gender, and healthy people/patients).

The effect of WBV on metabolism is interesting. The studies conducted so far on changes in metabolism under the influence of WBV mainly concern the influence simultaneously applying physical exercise and WBV. This type of exercise is known as vibration exercise. It has been shown that exhaustive whole-body vibration exercise elicits a mild cardiovascular exertion [16]. Milanese et al. [17] demonstrated that vibration can significantly increase the metabolic cost of exercise in a 20 min WBV training session. In another study, it has been demonstrated that the simultaneous use of WBV and exercise can significantly increase oxygen uptake (VO_2_) [18].

Vibration platforms, on which the subject stands, are usually used in studies assessing the physiological responses during WBV [14]. In WBV using vibrating plates, anti-gravity muscles are highly stimulated [19], as confirmed by increased electrical activity in electromyography (EMG) [20]. During vibration, muscle activity is necessary to suppress the vibration waves [19]. This is confirmed by studies in which the influence of vibration on balance has been reported [21,22]. Whole-body vibration can influence balance even when the displacement of the centre of gravity is small [19]. Thus, increased activity of core muscles plays a significant role in stabilising the body during WBV [19] and contributes to the increase in metabolic cost of exercise combined with WBV or comparing WBV to the control group (without WBV). In a manual by Rittwerger [16], it was indicated that the skeletal muscles are responsible for the increase in VO_2_. In a small number of studies, it has been suggested that WBV shifts energy metabolism towards carbohydrate utilisation and that this may increase post-exercise oxygen uptake. However, more studies are needed before drawing conclusions [16].

To date, the effect of WBV on resting metabolic rate (RMR) has not been established. Based on previous studies, it can be hypothesised that WBV should also affect RMR. As stated earlier, the effects of WBV were reported to include an increase in body (skin) temperature blood flow and muscle activity, suggesting a rise in metabolism. However, the use of vibration platforms and the assessment of the metabolic cost in standing position, during which the postural muscles are active to suppress the waves of vibrations, did not allow for the assessment of RMR or substrate utilisation at rest. The evaluation of the effects of WBV on RMR requires, first of all, the application of WBV in a lying position to inactivate the postural muscles. To our knowledge, this is the first study in which WBV was performed in the prone position and the first time that acute effects on RMR were assessed in young healthy women.

The aim of the research was to determine the acute effects of single-session vibration on resting metabolic rate and carbohydrate–lipid profile of blood in young, healthy women. It was hypothesised that WBV increases resting metabolic rate in healthy women.

## 2. Methods

### 2.1. Study Design

**Figure 1:**
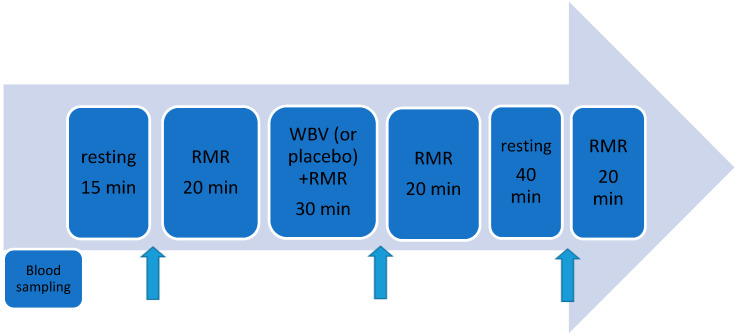
Measurement diagram for whole-body vibration and placebo condition (random order of groups). RMR—resting metabolic rate, WBV—whole-body vibration.

Young healthy women, in a randomised controlled crossover study, participated in 2 trials. During one visit to the laboratory, a vibration treatment (WBV) was performed, and during another, a vibration simulation (placebo-PL) was applied. The tests were carried out at a monthly interval, always between the 5th and 10th day of the menstrual cycle (in follicular phase). The RMR measurement was performed 4 times: (I) immediately before the vibration (measurement lasting 20 min), (II) throughout the duration of the vibration (30 min), (III) immediately after the completion of vibration (measurement lasting 20 min), and (IV) 1 h after the completion of the vibration (measurement lasting 20 min) (Figure 1). Participants were maintained in lying position throughout the study. The first RMR measurement was taken after a 15 min rest. Between the 3rd and 4th RMR measurements, the women remained in supine position and rested. For biochemical analyses, venous blood was collected in supine position: before the beginning of the study (prior to the first RMR measurement), after a 15 min rest, and immediately after the study and 1 h after the end of the WBV or PL procedure (Figure 1).

All the study participants were acquainted with the objectives of the study as well as the procedures, and provided their written consent to participate in the study. The research protocol was approved by the Bioethics Committee at the District Medical Chamber in Kraków (opinion No. 48/KBL/OIL/2018).

### 2.2. Participants

For the study, we recruited 13 healthy women aged 20–25 (21.6 ± 1.7 years) with a normal menstrual cycle (25–35 days). The study excluded women using hormonal contraceptives, menstruating irregularly or with other menstrual disorders. To rule out contraindications for participation in the trials, the participants underwent the following tests: morphology, glucose, glycosylated haemoglobin and ECG. Based on the results of these tests, an interview and physical examination, a physician assessed the participants’ health state and qualified for them for participation in the project. The women made a commitment not to change their diet or physical activity during the study period. The average body mass of the examined women was 57.7 ± 9.9 kg, and height totalled 164.5 ± 5.5 cm. The body mass of the participants did not change significantly between the measurements (WBV and PL) (*p* > 0.05).

### 2.3. Somatic Measurements

Body height was measured with a stadiometer (Seca, Germany) to the nearest 1 mm, while body mass was calculated using the Jawon Medical scale (IOI-353, Korea) to the nearest 0.1 kg.

### 2.4. Resting Metabolic Rate

The RMR measurement was performed in fasting state, always at the same time of the day (morning), between 6.00–10.00 a.m. Participants were informed to take the test fully rested, to avoid physical exertion for 2–3 days before the measurement, to be properly hydrated, not to change their eating habits or current level of physical activity between the measurement sessions and not to use any stimulants (nicotine) prior to the measurements. The RMR measurement was performed in prone position in an air-conditioned vibration therapy laboratory. The laboratory was kept at a constant temperature of 21 °C. Resting metabolic rate was measured by indirect calorimetry (breath-by-breath measurement) using the Cortex MetaLyzer 3R ergospirometer (Germany). Each time, the ergospirometer was calibrated in accordance with the manufacturer’s guidelines (gas and volume calibration). During the trial, the following were measured: VO_2_, carbon dioxide production (VCO_2_), respiratory quotient (RQ), resting metabolic rate, substrate utilisation (g/h) and energy expenditure (EE in kcal/h) from each substrate (carbohydrates—CHO, fats—FAT, proteins—PRO). RMR is expressed in absolute terms (kcal/day) and relative to body mass (kcal/kg/day) as well as body surface area (RMR/BSA) (kg/m^2^/day). Analysis of the results took into account the mean values of RMR and other indicators measured in indirect calorimetry from the 5 min measurement period, during which the steady state in oxygen uptake was detected. Steady state was considered to be fluctuations in oxygen uptake within the range of ±10%. The averages from steady state were used to calculate REE using the Weir [23] formula without using urinary urea nitrogen. All calculations were performed using dedicated metabolic rate measurement software provided by ergospirometer producer (Cortex, Germany).

### 2.5. Biochemical Analysis

Blood samples were collected in fasting state from the antecubital vein (BD Vacutainer^®^ vacuum system, Franklin Lakes, NJ, USA), after approximately 8 h of sleep (last meal no more than 2 h before bedtime). The following were determined in the blood: glucose concentration (GLU), ketone bodies (KB) (β-hydroxybutyrate—BHB), triglycerides (TG) and free fatty acids (FFA). GLU concentration was determined in the plasma (EDTA and glycolysis inhibitors: sodium fluoride and potassium oxalate). Triglycerides (TG), FFA and BHB, were determined in the serum (clotting activator). The assays were carried out using commercial reagent kits according to the procedure indicated by the manufacturers.

The detection range was, respectively: 0.11–41.6 mmol/L for glucose (GLUC3 Roche Diagnostics International Ltd., Germany), 0.07–2.24 mmol/L for FFA (NEFA FA 115, Randox Laboratories Ltd., UK), 0.1–5.75 mmol/L for 3-hydroxybutyrate (Ranbut, Randox Laboratories Ltd., UK) and 0.11–5.93 mmol/L for triglycerides (TRIG, Ortho-Clinical Diagnostics, France). The determinations were performed using the Cobas c 701/702 (glucose) and Cobas Bio (FFA, BHB) analysers by Roche Diagnostics International Ltd. (Germany) and Vitros 5.1 FS (TG) by Ortho-Clinical Diagnostics (France). The intra-assay % coefficients of variation were: KB 5.2%, TG 1.1%, FFA 4.3%, GLU 1.1%.

### 2.6. Whole-Body Vibration

**Figure 2:**
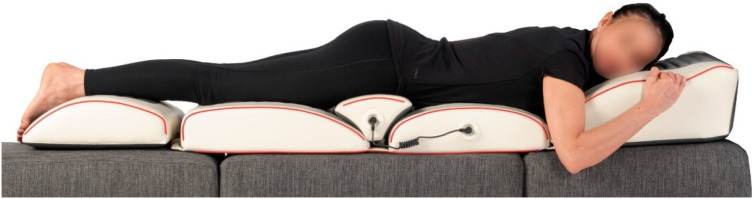
The position of the body on the device generating the vibration stimulus (illustrative photo).

In the study, cycloidal-oscillatory vibration was used. The intervention was performed in prone position with the use of a RAM Vitberg+ Base Module (active medical device, class IIa) enhanced with a RAM Vitberg+ Metabolism module (active medical device, class I) (Vitberg, Poland). The whole RAM Vitberg+ system combined oscillations of a general nature (whole-body vibration using the base module, covering the area of the trunk, upper limbs and thighs) with additional local action (Metabolism module) aimed at the following areas: epigastrium, mid-abdomen, pubic region, inguinal region, lateral and hypochondriac regions of the abdomen (Figure 2). The physical stimulus comprised vibrations generated in 3 perpendicular directions (3D), causing intermittent pulsations with variable values of frequency, amplitude and acceleration, which were within the range of: 25–52 Hz, 0.1–0.5 mm and 6.9–13.5 m/s^2^. The exposure to vibration lasted 30 min.

During the placebo treatment, the same device was used (vibration mattress), which did not generate vibrations, but only the sound identical to the vibration. Participants were informed that during this procedure, imperceptible vibrations of very low amplitude, frequency and acceleration are generated. The test procedure and body position were the same as in the case of vibration.

### 2.7. Statistical Analysis

Data distribution was checked using the Shapiro–Wilk test. All variables presented a normal distribution. Analysis of variance (ANOVA) with repeated measures was used to analyse the obtained data. The influence of the type of treatment (vibration vs. placebo), changes over time (baseline, treatment, post) and interactions between the main factors were assessed. In the case of a significant influence of the main factor (ANOVA, *p* < 0.05), post hoc analysis was performed using Fisher’s LSD test. Additionally, in post hoc analysis, the effect size (Cohen’s d) between baseline and WBV (placebo) treatment was calculated and interpreted as small (0.20), medium (0.50) or large (0.80) [24]. Data are presented as mean values and standard deviations. In the graphs, the vertical bars represent the 95% confidence interval. The STATISTICA 13 package (StatSoft, Inc., Tulsa, OK, USA) was used for calculations.

## 3. Results

**Figure 3:**
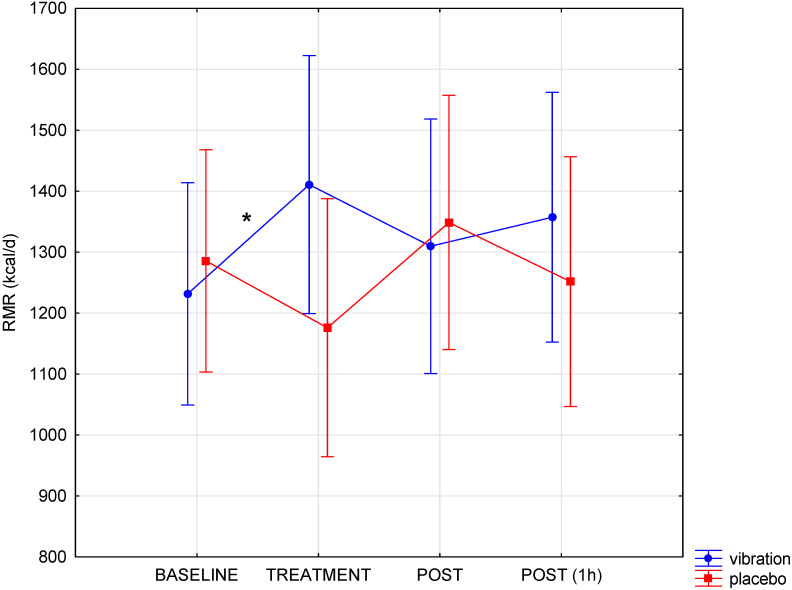
Changes in resting metabolic rate (RMR) pre- (baseline), during (treatment) and post-vibration (* significant change compared to baseline in WBV group). The data are presented as mean and 95% CI.

**Figure 4:**
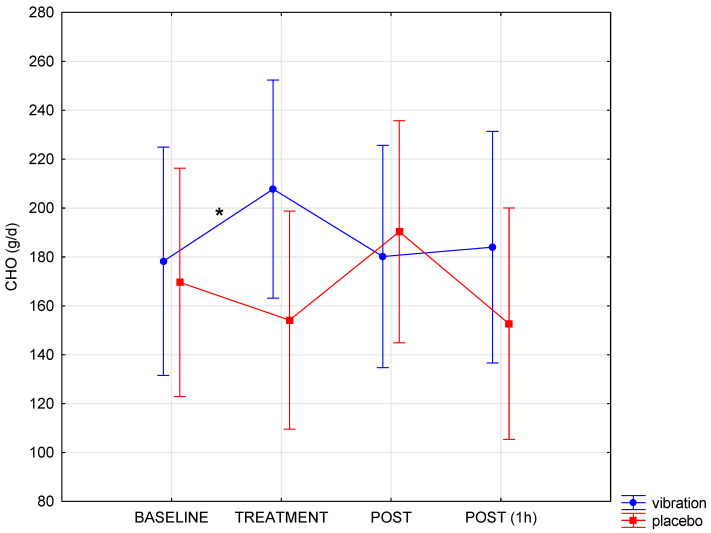
Changes in carbohydrate (CHO) utilisation pre- (baseline), during (treatment) and post-vibration (* significant change compared to baseline in WBV group). The data are presented as mean and 95% CI.

During the trial, no side-effects of the vibration were reported. The obtained results allow us to indicate a different course of changes in VO_2_, RMR, and CHO oxidation during WBV and in the placebo condition (interaction condition x time, *p* < 0.05; Table 1). During WBV, there was a significant increase in oxygen uptake and resting metabolic rate compared to baseline (Table 1, Figure 3). This effect only occurred in the case of the WBV procedure. Immediately after and 1 h following the end of the procedure, WBV, VO_2_ and RMR were close to baseline levels (*p* > 0.05). The increased energy expenditure resulted from the increased utilisation of carbohydrates and proteins during the vibration (Table 1, Figure 4). In the placebo condition, there were no significant changes over time in the level of the studied indices during calorimetry. The applied vibration had no significant effects on the level of glucose in the blood and the level of lipid markers (Table 2). All biochemical indicators, in each measurement, were within the range of physiological norms for the age and sex of the examined participants. The size of the observed effects should be considered medium to large (Table 1)

## 4. Discussion

Since, to the best of our knowledge, WBV in the prone position has not yet been used to evaluate metabolic response, full interpretation of the data is difficult and the only benchmark is WBV on a vibration platform while standing, often combined with exercise. Furthermore, the metabolic response to WBV differs according to participants’ fitness level, exercise type and vibration frequency [25]; therefore, the reported results of previous studies are not consistent. The aim of the study was to determine the acute effects of WBV on resting metabolic rate and substrate utilisation in young, healthy women in the follicular phase of menstrual cycle. In this research, a significant increase in oxygen uptake and RMR was noted in the tested women during vibration. This effect lasted only during the duration of the vibration and dissipated immediately after its completion. There were no changes in the tested parameters 1 h after the end of the vibration (they were close to the baseline). The side effects were also not reported. In the placebo group, a similar increase in VO_2_ or RMR during the vibration simulation was not observed. Rittwerger [16] has suggested that WBV (with the concomitant use of exercise and vibration) shifts energy metabolism towards carbohydrate utilisation and that it may increase post-exercise oxygen consumption. The results of this study do not confirm the statement that WBV influenced oxygen consumption following the treatment—the effect of increased oxygen consumption was maintained only during WBV. Thus, this allows us to indicate that the cause of the increased oxygen uptake after vibration exercise is related to vibration platform exercise (increased intensity) rather than vibration *per se*. Moreover, Rittwerger [16] suggested that the observed effect of increased energy expenditure during WBV is quite moderate and probably irrelevant to long-term energy balance.

The novelty of this study is that, for the first time, whole-body vibration was used in the lying position, which allowed the RMR to be measured. As a result, the activity of core muscles and their response to possible balance changes caused by WBV was eliminated. This study indicates that increased oxygen uptake may not only be associated with increased muscle activity related to vibration wave suppression and correction of body posture for vibration-induced imbalances. Rosenberger et al. [26] demonstrated that resistive vibration exercise permanently elevated metabolic energy turnover, although the initially observed additional motor unit activity by vibration exercise could not be preserved in the working musculature. The response of the muscle to vibration stimulation is a tonic vibration reflex [19]. During vibration of the body, the skeletal muscles undergo small changes in muscle length. Burke et al. [27] suggested that the vibration reflex operates predominantly or exclusively on alpha motoneurons and does not use the same cortically originating efferent pathways as those used in the performance of voluntary contractions [28]. In the present study, no EMG recordings were performed, so further testing is needed to confirm muscle activity during WBV in the lying position with EMG. However, Zange et al. [29] reported the relaxed and unloaded calf muscles did not respond to vibration with a remarkable reflex contraction and the acceleration by vibration apparently only ejected capillary venous blood from the muscle. This suggests that the increased metabolism during WBV may not be related to reflex muscle activity but may be the cumulative response of several physiological mechanisms to WBV.

Fares et al. [30] indicated that substrate oxidation did not change in response to WBV. The results of this study indicate that the increase in RMR was associated with increased carbohydrate utilisation. At the same time, this did not lead to a reduction in blood glucose levels, but it confirms that blood glucose homeostasis was preserved. A slight decrease in blood glucose levels under the influence of WBV was reported by di Loreto et al. [31]. Our data also contradict the data presented by Licurci et al. [32], who showed that healthy elderly individuals subjected to whole-body vibration presented reduction in blood glucose levels. So, perhaps the vibration effect may vary in young and older people. Bunker et al. [33] demonstrated different effects of WBV in people below and above the age of 45: in comparison to younger ones, participants older than 45 years did not significantly improve in power measures after WBV. Perhaps the response to WBV is related also to fitness level [25]. The vibration used in our research did not affect the oxidation of fats. This is confirmed by previous studies in which WBV (performed on a vibration platform) did not affect fat oxidation across any vibratory loads [18]. Moreover, Kang et al. [34] reported no differences in heart rate or rates of carbohydrate and fat oxidation during either the WBV treatment or the subsequent exercise. Although a significant effect of a single WBV procedure on FFA metabolic rate and blood concentration was not demonstrated in our study, Goto and Takamatsu [35] found that 10 sessions of WBV increased FFA concentration during the recovery period but serum glycerol did not significantly change.

The physiological response may depend on the characteristics of the vibration used (frequency, amplitude, and acceleration) [16,24]. During exercise on a vibration platform, VO_2_ was higher in comparison to exercise without WBV, regardless of the applied frequency used. The increased metabolic effect of WBV seems load-dependent as WBV with an amplitude smaller than 2 mm did not elevate VO_2_ significantly [34].

The metabolic effects of WBV may differ depending on the type of vibration, i.e., local vs. WBV [19]. By subjecting the body to vibration, not only the muscles undergo vibration, but also the internal organs. This study involved women; they are characterized by greater body fatness than men, so it was expected that the effects of vibration on visceral fat might be more noticeable in women than in men. In our research, we used an additional local vibration module, the vibrations of which were directed towards the abdominal cavity. It was expected to see the effects of this vibration on visceral fat, i.e., changes in fat oxidation and in the level of blood lipids. However, the applied additional local vibration did not provide such an effect. In our study, blood TG levels decreased regardless of whether WBV or placebo treatments were used, which may be the result of prolonged meal-time intervals, their hydrolysis and the need to compensate for plasma FFA levels.

Metabolic rate is affected by hormones, including those of the menstrual cycle. Basal metabolic rate changes steadily throughout the cycle and decreases during menstruation, falling to its lowest point approximately 1 week before ovulation, and subsequently rising until the beginning of the next menstrual period [36]. In previous studies, it has been reported that men and women respond similarly to a vibratory stimulus despite the difference in body mass [34]. This trial is, to our knowledge, the first to include menstrual cycle in the assessment of resting metabolic rate during WBV—the women were in the follicular phase of the menstrual cycle, i.e., between the 5th and 10th day of the menstrual cycle. In other studies, conflicting data have been reported about the effects of WBV on hormonal response. Erskine et al. [37] demonstrated no significant changes in salivary concentration of testosterone or cortisol suggesting that the WBV was of low acceleration and concluded that WBV does not represent a stressful stimulus for the neuroendocrine system. The concentrations of plasma epinephrine and norepinephrine increased immediately after the WBV session [35], indicating that these hormones may have significantly influenced metabolism. On the other hand, Bosco et al. [38] suggested that both the hormonal responses to WBV (increase in testosterone and growth hormone concentration and a decrease in cortisol concentration) and the increase in neuromuscular effectiveness were simultaneous but independent, and may have common underlying mechanisms of performance improvement. Opposite effects were presented by Cardinale et al. [39], showing that a single session of WBV exposure does not noticeably alter serum testosterone or IGF-1 levels. Hormonal responses to WBV (insulin, glucagon, cortisol, epinephrine, norepinephrine, growth hormone, IGF-1, free and total testosterone), with the exception of norepinephrine, are not affected by acute vibration exposure [31].

Increased metabolism during WBV may also be the result of changes in circulation in the vibrated tissue and changes in tissue (body) temperature [40]. An increase in mean blood flow was observed under the influence of vibrational stimulation compared to the placebo group [9]. Robbins et al. [40] points to a high level of sensitivity of the peripheral vascular system to vibration exposure. Acute WBV elevates muscle temperature more quickly than traditional forms of cycling and passive warm-up [41], but the thermogenic effect of intermittent WBV, whilst robust, was quantitatively small (<2 METS) [30].

## 5. Limitation of the Study

In this study, the acute effects of WBV performed in the prone position on resting metabolic rate and substrate utilisation were evaluated. In this research, increased oxygen uptake and RMR during WBV were demonstrated. In previous studies, increased oxygen uptake during WBV was attributed precisely to skeletal muscles. In this research, the activity of postural muscles and increased muscle work aimed at maintaining balance during WBV was eliminated. The results indicated the need for further research to establish the physiological mechanisms underlying the observed effects of WBV on resting metabolic rate, e.g., changes in hormones that affect metabolism, tissue temperature, blood flow, or the activity of muscles relaxed due to lying body position (EMG) into account. However, we did not investigate this in our study.

Another limitation of the study is the relatively small number of participants (healthy, young women in the follicular phase of the menstrual cycle), and the fact that the effects of only one type of vibration with specific characteristics (duration, amplitude, and frequency) were studied.

## 6. Conclusions

In this research, a significant increase was demonstrated in oxygen uptake, and thus, in resting metabolic rate, when applying body vibration in the prone position in young, healthy women in the follicular phase of menstrual cycle. Analysis of the utilisation of substrates in calorimetry showed that the increase in RMR was due to the increase in glucose utilisation during vibration. At the same time, we did not note any significant decreases in blood glucose levels following vibration. The applied vibration did not significantly affect the concentration of the analysed lipid indices, which were within the physiological norms for all measurements.

## Figures and Tables

**Table 1:** Effects of vibration on variables measured during calorimetry.

Variable	Mode	BASELINE	TREATMENT	POST	POST (1 h)	Effect: Mode F (*p*)	Effect: Time F (*p*)	Interaction F (*p*)	*p*: Post Hoc [Cohen’s d]
		I	II	III	IV				
VO_2_ (L/min)	WBV	0.17 ± 0.04	0.20 ± 0.05	0.19 ± 0.05	0.19 ± 0.05	0.35 (0.62)	0.67 (0.57)	3.04 (0.03)	I–II:0.02 [0.67]
	PL	0.18 ± 0.04	0.16 ± 0.04	0.19 ± 0.05	0.18 ± 0.04				NS
VCO_2_ (L/min)	WBV	0.15 ± 0.04	0.18 ± 0.04	0.16 ± 0.04	0.17 ± 0.04	0.02 (0.90)	2.40 (0.07)	1.52 (0.21)	NS
	PL	0.15 ± 0.04	0.14 ± 0.04	0.16 ± 0.05	0.15 ± 0.04				NS
RQ	VIBR	0.87 ± 0.05	0.88 ± 0.03	0.86 ± 0.03	0.87 ± 0.05	0.27 (0.61)	1.46 (0.23)	2.55 (0.06)	NS
	PL	0.85 ± 0.07	0.86 ± 0.06	0.86 ± 0.06	0.83 ± 0.06				NS
RMR (kcal/kg/day)	WBV	21.8 ± 6.6	25.1 ± 7.6	23.4 ± 7.3	23.8 ± 7.3	0.14 (0.70)	0.67 (0.57)	2.12 (0.10)	NS
	PL	22.2 ± 6.4	19.8 ± 5.9	23.2 ± 7.6	21.6 ± 7.5				NS
RMR/BSA (kg/m^2^/day)	WBV	772.1 ± 222.2	889.2 ± 262.9	824.2 ± 237.4	843.1 ± 246.1	0.24 (0.62)	0.62 (0.60)	3.18 (0.02)	I–II: 0.01 [0.48]
	PL	785.6 ± 205.1	701 ± 223.1	821.6 ± 248.5	759.3 ± 236.3				NS
FAT (g/h)	WBV	49.2 ± 24.8	55.2 ± 24.9	57.3 ± 18.7	58.5 ± 30.4	0.07 (0.79)	1.17 (0.59)	0.59 (0.62)	NS
	PL	63.4 ± 37.4	48.7 ± 31.1	62 ± 30.8	64.9 ± 31.3				NS
PRO (g/h)	WBV	14.2 ± 3.7	16.1 ± 4.6	15.1 ± 3.9	15.3 ± 4.1	0.22 (0.63)	0.50 (0.68)	2.61 (0.06)	NS
	PL	14.1 ± 3.5	12.7 ± 3.9	14.9 ± 4.21	13.9 ± 3.6				NS
EE_CHO (kcal/h)	WBV	30.6 ± 13.0	36.1 ± 10.9	30.7 ± 11.7	31.9 ± 11.04	0.50 (0.48)	1.37 (0.25)	4.83 (0.004)	I–II:0.02 [0.46] II–III:0.03
	PL	25.6 ± 14.9	25.9 ± 15.3	28.7 ± 15.1	23.1 ± 16.6				NS
EE_FAT (kcal/h)	WBV	19.2 ± 9.6	21.3 ± 9.6	22 ± 7.4	22.4 ± 11.9	0.08 (0.76)	1.07 (0.36)	0.52 (0.67)	NS
	PL	24.6 ± 14.4	19 ± 12.1	24 ± 11.9	25.1 ± 12.3				NS
EE_PRO (kcal/h)	WBV	2.3 ± 0.7	3 ± 0.89	2.4 ± 0.76	2.5 ± 0.87	0.13 (0.71)	0.74 (0.53)	3.65 (0.01)	I–II:0.004 [0.88] II–III:0.01 II–IV:0.04
	PL	2.3 ± 0.6	2.1 ± 0.7	2.6 ± 0.9	2.4 ± 0.7				NS

WBV—whole-body vibration; PL—placebo; VO_2_—oxygen uptake; VCO_2_—carbon dioxide production; RQ—respiratory quotient; RMR—resting metabolic rate; BSA—body surface area; CHO—carbohydrates; PRO—proteins; EE—energy expenditure; ES—effect size. The results are presented as means and SD. NS—Not Statistically Significant.

**Table 2:** Effects of vibration on biochemical variables.

Variable	Mode	BASELINE	POST	POST (1h)	Effect: Mode F (*p*)	Effect: Time F (*p*)	Interaction F (*p*)	*p* (Post Hoc)
		I	II	III				
GLU (mmol/L)	WBV	5.11 ± 0.52	4.96 ± 0.32	4.86 ± 0.32	0.43 (0.52)	2.72 (0.08)	0.02 (0.97)	NS
	PL	4.99 ± 0.70	4.81 ± 0.38	4.78 ± 0.37				NS
TG (mmol/L)	WBV	0.82 ± 0.31	0.73 ± 0.28	0.69 ± 0.25	0.005 (0.94)	11.59 (<0.001)	0.46 (0.63)	I–II: 0.04 I–III:0.002
	PL	0.83 ± 0.36	0.70 ± 0.30	0.70 ± 0.24				I–II: 0.002 I–III:0.003
FFA (mmol/L)	WBV	0.42 ± 0.22	0.37 ± 0.41	0.40 ± 0.29	0.41 (0.52)	5.26 (0.009)	2.87 (0.06)	NS
	PL	0.54 ± 0.25	0.32 ± 0.23	0.58 ± 0.38				I–II:0.003 II–III: <0.001
KB (mmol/L)	WBV	0.04 ± 0.03	0.04 ± 0.04	0.06 ± 0.08	0.69 (0.41)	2.63 (0.08)	0.54 (0.58)	NS
	PL	0.10 ± 0.13	0.07 ± 0.11	0.12 ± 0.23				NS

WBV—whole-body vibration; PL—placebo; GLU—glucose; TG—triglyceride; FFA—free fat acids; KB—ketone bodies. The results are presented as means and SD. NS—Not Statistically Significant.

## Data Availability

The datasets used and/or analysed during the current study are available from the corresponding author on reasonable request.
